# Anatomical organization of forebrain circuits in the primate

**DOI:** 10.1007/s00429-022-02586-8

**Published:** 2022-10-21

**Authors:** Franco Giarrocco, Bruno B. Averbeck

**Affiliations:** Laboratory of Neuropsychology, National Institute of Mental Health, National Institutes of Health, Building 49 Room 1B80, 49 Convent Drive MSC 4415, Bethesda, MD 20892-4415 USA

**Keywords:** Cortex, Striatum, Thalamus, Vertebrate, Organizational principles, Forebrain evolution

## Abstract

The primate forebrain is a complex structure. Thousands of connections have been identified between cortical areas, and between cortical and sub-cortical areas. Previous work, however, has suggested that a number of principles can be used to reduce this complexity. Here, we integrate four principles that have been put forth previously, including a nested model of neocortical connectivity, gradients of connectivity between frontal cortical areas and the striatum and thalamus, shared patterns of sub-cortical connectivity between connected posterior and frontal cortical areas, and topographic organization of cortical–striatal–pallidal–thalamocortical circuits. We integrate these principles into a single model that accounts for a substantial amount of connectivity in the forebrain. We then suggest that studies in evolution and development can account for these four principles, by assuming that the ancestral vertebrate pallium was dominated by medial, hippocampal and ventral–lateral, pyriform areas, and at most a small dorsal pallium. The small dorsal pallium expanded massively in the lineage leading to primates. During this expansion, topological, adjacency relationships were maintained between pallial and sub-pallial areas. This maintained topology led to the connectivity gradients seen between cortex, striatum, pallidum, and thalamus.

## Introduction

The primate forebrain, composed of the cortex, basal ganglia, and thalamus, is one of the most complex structures in biology. Using various parcellation schemes, the macaque cortex can be divided into as many as 150 unique areas (Morris et al. 2000; Reveley et al. 2016; Saleem and Logothetis 2012; Van Essen and Glasser 2018), each area projects to 10 s of other areas, and thus there are 1000 s of identified anatomical connections just within the cortex. Once one considers connections between cortex, basal ganglia, and thalamus, the complexity increases. It may be possible, however, to find principles that simplify this complexity. Such principles can also be used to suggest hypotheses for how the forebrain gives rise to behavior.

Previous authors have identified several principles that simplify and clarify our understanding of forebrain organization. The principles all follow from consideration of patterns in anatomical connectivity, identified with modern tract tracing methods. The principles include cortical–basal ganglia–thalamocortical loops (Alexander et al. 1986; Haber 2016; Middleton and Strick 2000), a nested organization of connectivity among neocortical areas (Averbeck et al. 2009; Giarrocco and Averbeck 2021), a dorsal/ventral organization of visual processing (Ungerleider and Mishkin 1982), preferential connectivity between areas with similar architectonic differentiation in prefrontal cortex (Barbas and Rempel-Clower 1997; García-Cabezas and Zikopoulos 2019; Goulas et al. 2018), matching patterns of sub-cortical connectivity between connected cortical areas (Selemon and Goldman-Rakic 1988; Yeterian and Van Hoesen 1978), layer specific connections that define feed-forward vs. feedback connections (Felleman and Van Essen 1991; Rockland and Pandya 1979), a dual origin pattern to the organization of frontal cortical areas (Pandya and Yeterian 1985; Sanides 1970) and triple descending connections from cortex through the striatum, pallidum, and thalamus that ultimately terminate in brain stem areas that control behavior (Swanson 2000). Each of these principles provides a way to reduce the complexity of forebrain connectivity, by substituting a simplified model for a large amount of raw anatomical data.

Our goal in this review is to synthesize and extend previous models that have accounted for the anatomical connectivity of the forebrain into a single framework that attempts to account for the large-scale organization of most of the forebrain beyond early visual and auditory sensory areas. Although models always eliminate detail, and therefore information, they drive hypotheses and may account for much of the relevant structure. Thus, this model does not account for every anatomical connection in the forebrain. Rather it suggests a simplified architecture that accounts for the dominant patterns of connectivity. Some of the principles we put forward have been substantiated by statistical models (Averbeck et al. 2009, 2014; Averbeck and Seo 2008; Caminiti et al. 2017; Giarrocco and Averbeck 2021). As with any models, the goal is to build a simple model that accounts for as much variance as possible. Models, however, always make some assumptions, and always eliminate some information. Future work, perhaps with genetic or imaging methods that allow for more rigorous quantitative testing of these ideas (Burt et al. 2018; Gomez et al. 2019, 2021), may further support (or not) the ideas we put forth.

After presenting the model, we interpret the proposed organization in the context of work in evolution and development, as these fields offer an explanation for the organization that we propose. Much of the recent work in development focuses on brain organization and not anatomical connectivity (Puelles et al. 2017, 2019). However, we suggest that connectivity is related to the structural organization, and the structural organization provides a simplified model for the connectivity.

**Fig. 1:**
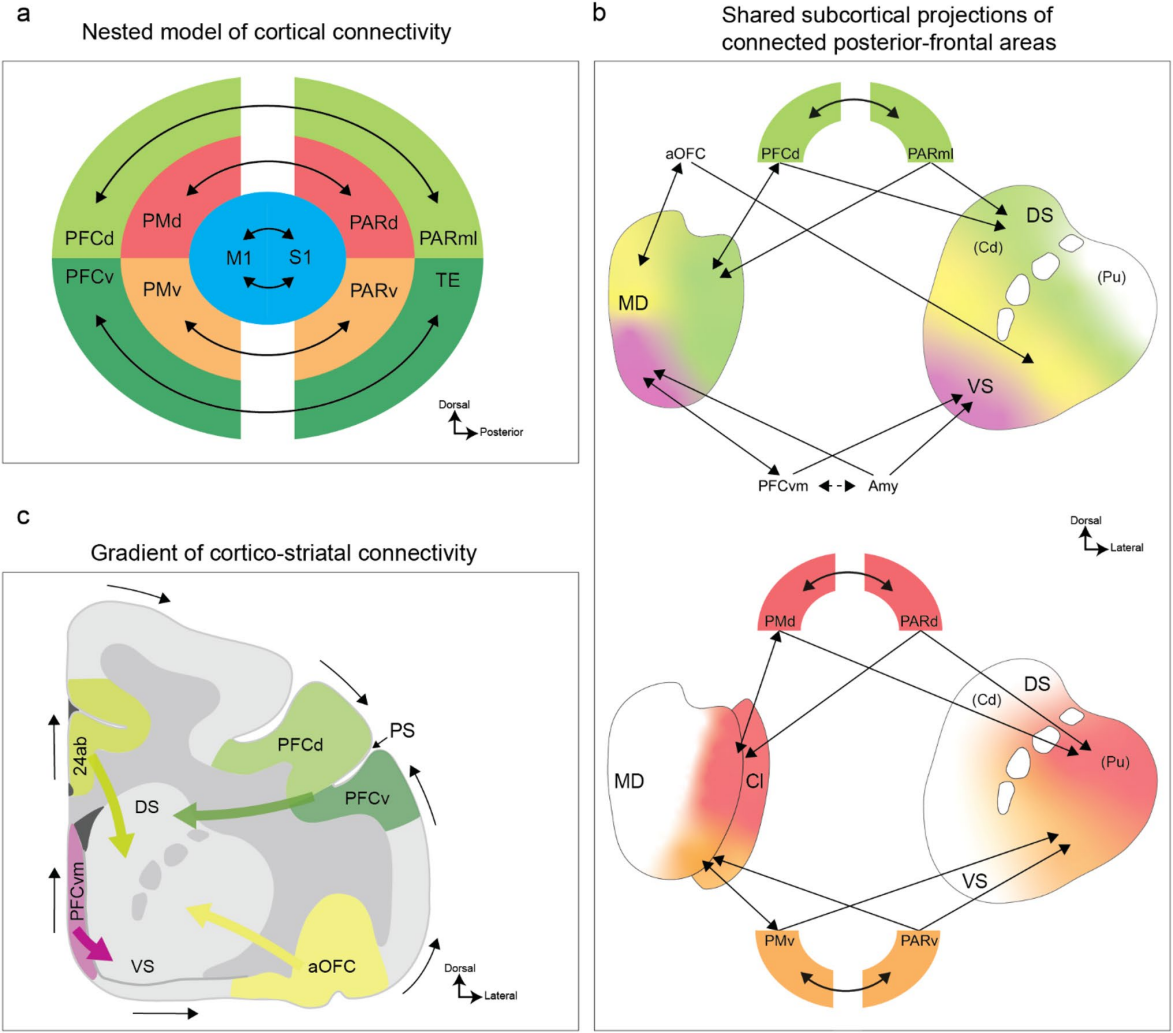
Principles of anatomical connectivity in the primate forebrain. **a** Schematic diagram of dominant circuits between parietal, temporal and frontal neocortical areas. These circuits outline a nested pattern of connectivity, with the M1-S1 circuit at its core. Outside of M1-S1, dorsally, circuits between dorsal parietal (PARd) and dorsal premotor (PMd) areas and, ventrally, between ventral parietal (PARv) and ventral premotor (PMv) areas define the next level. The outermost level of the nested pattern is defined by circuits between mediolateral parietal (PARml) and dorsal prefrontal (PFCd) areas, and between temporal cortex (TE) and ventral prefrontal cortex (PFCv). **b** Connected parietal, temporal, and frontal areas send overlapping projections to the striatum and the mediodorsal (MD) thalamus. **c** Topographic organization of connectivity between areas in prefrontal cortex and the striatum. PFCvm projects to the ventral striatum. Areas progressively more distant, either mediodorsally or ventrolaterally, project to more dorsal striatal regions. For example, the anterior cingulate cortex (24ab) and the anterior orbitofrontal cortex (aOFC) project to the middle striatum. PFCd and PFCv, which are adjacent to the principal sulcus (PS) project to the dorsal striatum (DS). Thus, the topographic organization of prefrontal inputs defines dorsal and ventral poles in the anterior striatum

We explain our model using four principles drawn from the literature (Fig. 1). The first principle we discuss is the nested model of parietal/temporal to frontal connectivity (Giarrocco and Averbeck 2021; Vijayakumar et al. 2019). This model follows from a consideration of the statistical organization of connectivity patterns between posterior (parietal and temporal) areas and frontal cortical areas. It suggests that these areas are connected in a nested pattern, most clear for neocortical areas (Fig. 1a). The second principle was originally put forward by Yeterian and Van Hoesen (Yeterian and Van Hoesen 1978) and later extended and refined by Selemon and Goldman-Rakic (Selemon and Goldman-Rakic 1988). This principle suggests that parietal and frontal or temporal and frontal areas that are connected, send connections to overlapping regions in the thalamus and striatum (Fig. 1b). For example, area Opt/7a in parietal cortex is connected to dorsolateral prefrontal cortex (PFCd), and these cortical areas send overlapping projections to the dorsal striatum and the lateral portion of the medial dorsal (MD) thalamus (Selemon and Goldman-Rakic 1985, 1988). Thus, connected nodes in the nested circuits have overlapping projections in sub-cortical structures. The third principle, apparent in the frontal–striatal connectivity shown by Haber et al. (Haber et al. 2006), and subsequently quantified with a model (Averbeck et al. 2014), is an ordered topographic arrangement of connectivity between locations in frontal cortex and the striatum (Fig. 1c). PFCd projects to the dorsal striatum and caudal orbitofrontal cortex (13/agranular insula) and ventromedial prefrontal cortex (PFCvm) project to the ventral striatum. These connections, therefore, define poles in the anterior striatum (Averbeck and Murray 2020) and areas between these cortical areas project to intermediate locations in the striatum. As one translates along the cortical surface from ventromedial prefrontal cortex, either dorsally or laterally, toward lateral prefrontal cortex, the projections into the striatum translate dorsally, from the ventral striatum to the dorsal striatum, in a monotonic way. Similar gradients can be identified in the MD and adjacent centro-lateral (CL) thalamus (Fig. 1b), with ventromedial and caudal orbital prefrontal areas projecting medially in the MD, and dorsolateral prefrontal cortex projecting laterally in the MD (Barbas et al. 1991; Goldman-Rakic and Porrino 1985). The fourth principal, which is related to the third principle, is the closed-loop, topographic organization of cortical–basal ganglia–thalamocortical circuits (Alexander et al. 1986). This principal shows that the topographic organization of cortical projections into the striatum continues through the globus pallidus internal segment and substantia nigra pars reticulata, to the thalamus, and back to cortex. By combining these four principles, one can account for a large fraction of the connectivity of the primate forebrain. Furthermore, we will suggest that these principles follow from the massive expansion of the cortex in mammals, particularly in primates, and the topological organization of connectivity. Our hypothesis is that cortex, striatum, pallidum, and thalamus expanded in a (mostly) concerted (Finlay 2009; Finlay and Darlington 1995; Yopak et al. 2010) and topological way, and connectivity across these four nodes reflects this.

## Nested model of cortical connectivity

**Fig. 2:**
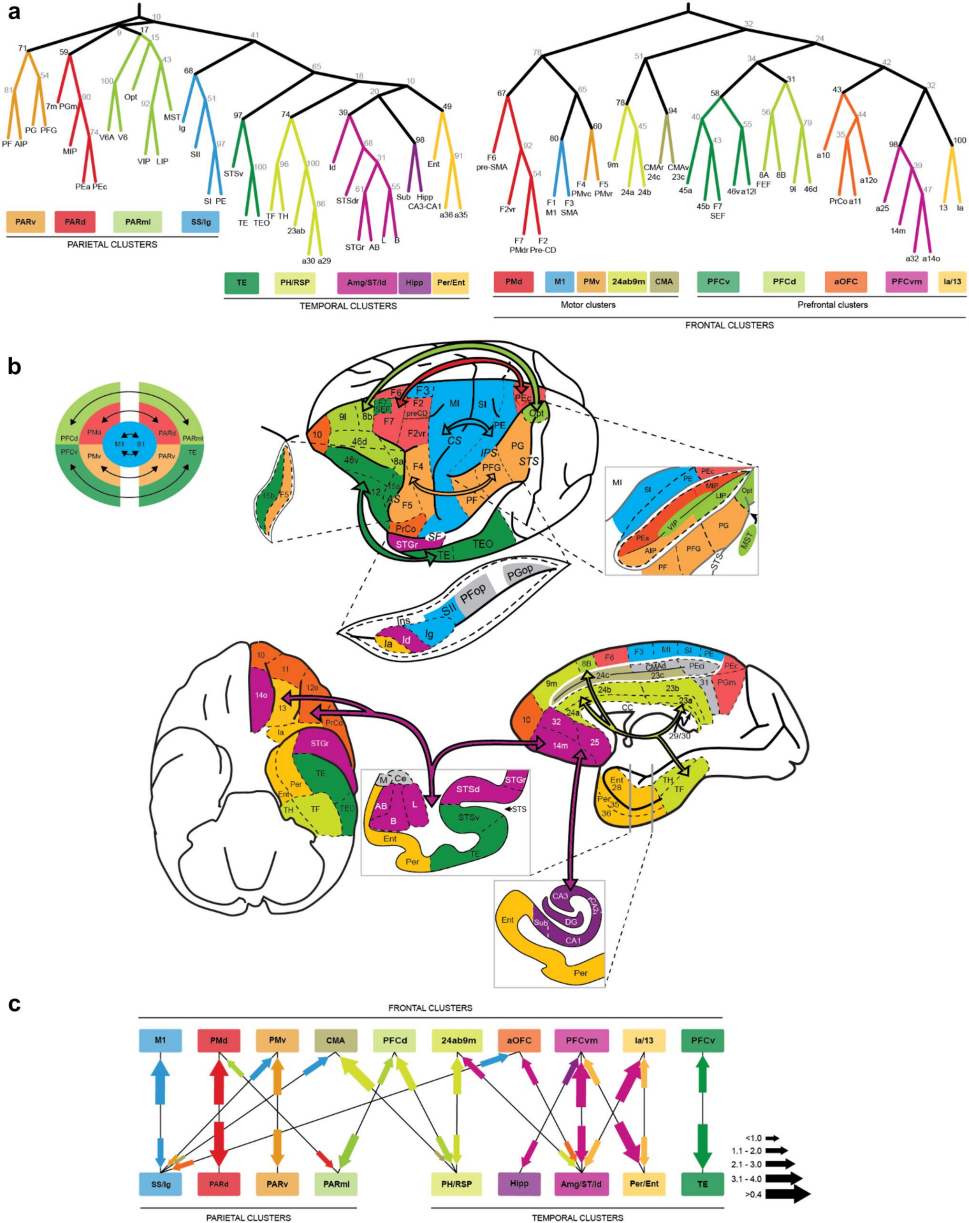
The nested model of cortical connectivity in the macaque monkey. **a** Clusters of cortical areas based on their anatomically defined inputs are shown for posterior areas (parietal and temporal areas) on the left, and for frontal areas on the right. The number at each branch indicates the strength of the cluster (scale from 1 to 100). **b** View of dominant circuits underlying the parieto-frontal and temporo-frontal networks shown on the macaque cortex. Clusters composed of similar colors represent nodes of parieto-frontal and temporo-frontal circuits (arrows). **c** Main reciprocal connections among frontal, parietal, and temporal clusters. Arrow size (color) indicates the strength (source) of inputs. Figure adapted from Giarrocco and Averbeck (2021)

The cortex is composed of a large number of architectonically defined areas, and these areas are massively interconnected. There are, however, large-scale organizational trends superimposed on the complex interactions within cortex. Several authors have carried out statistical analyses on the matrix of cortical connectivity (Hilgetag et al. 2000; Stephan et al. 2000). Some of these groups have focused on calculating graph theoretic quantities, to define network architectural features of cortical networks (Bullmore and Sporns 2009; Reijneveld et al. 2007). In our own work, we have focused on defining simplified organizational features of the connectivity in cortex, by clustering, or lumping together areas that share similar inputs (Caminiti et al. 2017; Giarrocco and Averbeck 2021), and then examining connectivity between clusters (Fig. 2a, c). This approach assumes that the function of a cortical area is defined in large part by its inputs, and therefore areas that have similar inputs likely have similar functions. These analyses have shown that clusters are formed by spatially contiguous areas (Fig. 2b). This is partially because connectivity tends to be stronger between nearby areas, and therefore nearby areas tend to project to each other (Averbeck and Seo 2008). Distant projections also tend to cross architectonic boundaries and therefore inputs to areas are often shared by several adjacent areas.

For posterior areas, the cluster analyses showed that parietal areas cluster with other parietal areas, and temporal areas cluster with other temporal areas (Fig. 2a). Thus, there is a large-scale distinction in the connectivity of parietal and temporal cortex, as suggested by previous functional work (Ungerleider and Mishkin 1982). Within the parietal cluster there are four subordinate clusters. These clusters represent the inferior parietal areas (PARv), the dorsal parietal areas (PARd), the dorsomedial spatial visual areas (PARml), and the somatosensory cortex (SS/Ig). Within the temporal cluster, which also includes the caudal cingulate, there are five clusters. These represent temporal lobe visual areas (TE), the para-hippocampal and retrosplenial areas (PH/RSP), the amygdala and nearby temporal pole and superior temporal sulcus areas (Amg/ST/Id), the hippocampus and associated structures (Hipp), and perirhinal and entorhinal cortex (Per/Ent).

Superordinate clusters in frontal areas also capture the known large-scale organization (Fig. 2a). Motor areas cluster together (M1/PMd/PMv), and the motor cluster is also within a larger cluster (note these are unrooted trees) that includes the anterior cingulate (24ab/9 m/CMA). There is also a cluster composed of all prefrontal areas. Within this superordinate cluster, caudal orbital areas (13/Ia) cluster with ventral–medial prefrontal areas (PFCvm), which then form a superordinate cluster with anterior orbitofrontal cortex (aOFC). The lateral prefrontal areas (PFCd and PFCv) form the next cluster.

Although the cluster analysis shows that there is a robust organizational structure to the parietal, temporal, and frontal areas, it provides limited topological insight beyond the fact that areas closer together tend to cluster together. If we extend the cluster analysis by asking which posterior cluster (i.e., posterior to the central sulcus) is most strongly connected to each frontal cluster (i.e., anterior to the central sulcus), and vice versa, we can define the dominant circuits linking posterior areas to frontal areas (Fig. 2b, c). This approach eliminates many important connections. However, it provides a simplified conception of connectivity within the cortex, which emphasizes the strongest connections. When we do this, we find that connectivity tends to be reciprocal (Giarrocco and Averbeck 2021). Thus, the posterior cluster that provides the dominant input to each frontal cluster also tends to receive its dominant input from the same frontal cluster (Fig. 2b, c). This is not surprising, since reciprocal connectivity is a feature of the connectivity of single cortical areas (Rockland and Pandya 1979). But it further shows that posterior and frontal areas are connected in well-defined circuits.

When we visualize the dominant connectivity patterns on the cortex we can see that there is a regular, nested organization of connectivity, with M1 and S1 at its core (Fig. 2b). As one moves outward from the M1/S1 circuit dorsally, there is a dorsal parietal to dorsal premotor (PARd-PMd) circuit. Ventrally, there is a ventral parietal to ventral premotor (PARv-PMv) circuit. Outside of these parietal–premotor circuits, there are two more parietal/temporal–prefrontal circuits. Dorsally, there is a dorsal–mediolateral parietal to dorsal prefrontal circuit (PARml-PFCd) and ventrally a temporal to ventral prefrontal (TE-PFCv) circuit. Similarly, on the medial wall, there is a circuit connecting the caudal cingulate areas to the anterior cingulate (PH/RSP-24ab/9 m). The posterior cingulate is also well connected to dorsal prefrontal areas (PH/RSP-PFCd).

On the ventral surface of the brain, there is a circuit centered around caudal orbital area 13 and the agranular insula, which are connected to entorhinal and perirhinal cortex (Per/Ent-Ia/13). Beyond these areas, there is a circuit that connects the anterior superior temporal sulcus, the amygdala and hippocampus with the ventromedial prefrontal cortex (Amg/ST/Id/Hipp-PFCvm). Thus, there is a highly structured topological arrangement of connectivity between posterior and frontal areas. The organization of projections into lateral prefrontal areas, has been described previously (Barbas 1988). Some of the areas are not easily fit within this scheme. Anterior OFC areas 10, 11 and 12o have heterogeneous inputs that are not dominated by a single posterior area, and OFC also does not provide the dominant input to any posterior cluster, although it does provide strong inputs to the Per/Ent, Amg/ST/Id and TE clusters.

**Fig. 3:**
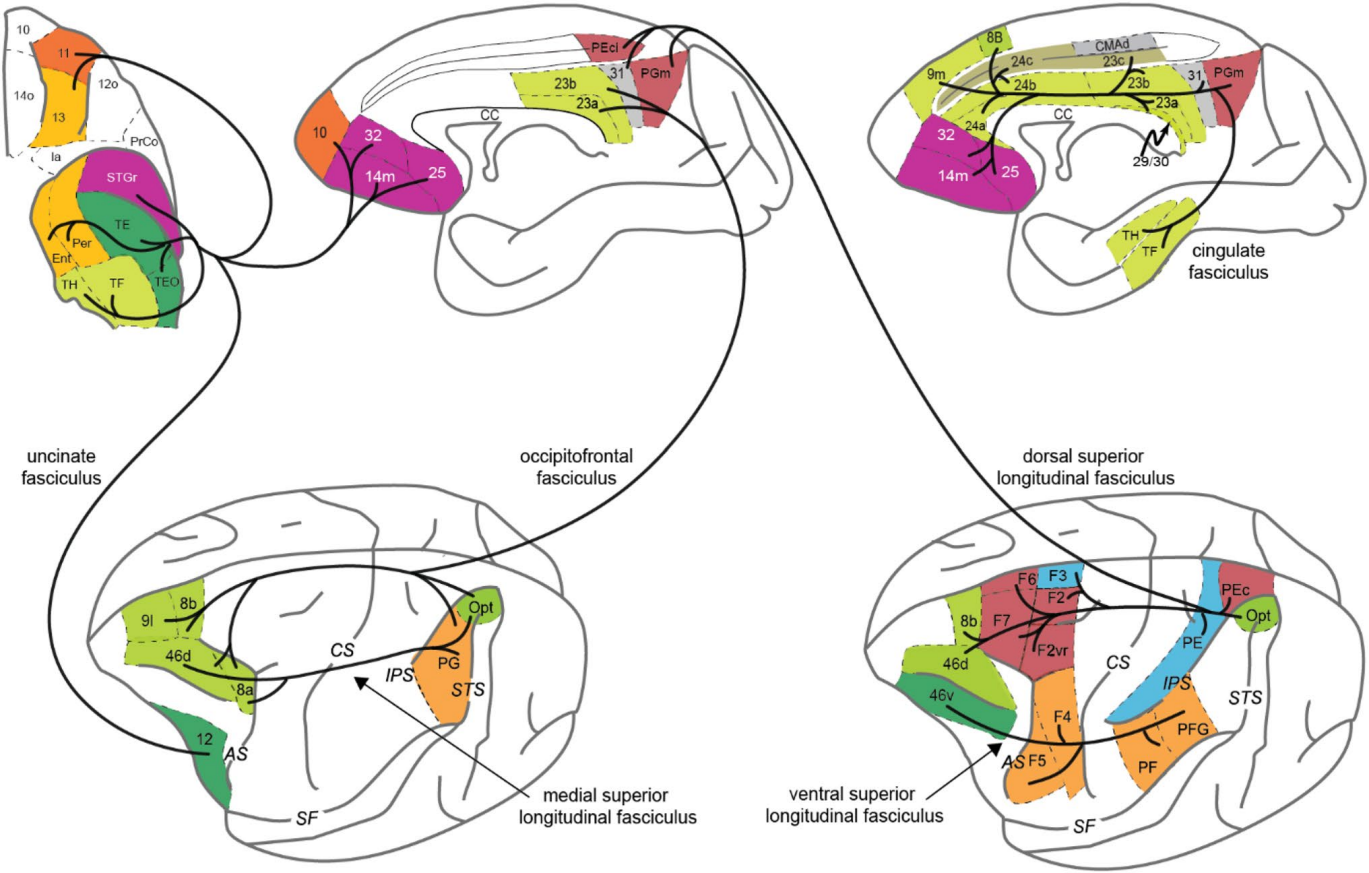
Organization of the main fiber bundles in the macaque cortex supporting the nested model of cortical connectivity. Sketch of the ventral (upper left panel), medial (upper middle and upper right panels), and dorsolateral (bottom panels) views of the macaque cerebral cortex. Areas connected by a given fiber bundle are colored according to the clustering shown in Fig. 2

The connectivity structure identified by the cluster analysis corresponds, approximately, to several of the main white matter tracts in cortex (Yeterian et al. 2012) (Fig. 3). The dorsal portion of the superior longitudinal fasciculus (dSLF) connects PARd to PMd and the ventral portion of the SLF (vSLF) connects PARv to PMv. The occipitofrontal fasciculus (OFF) and middle portion of the superior longitudinal fasciculus (mSLF) connect PARml to PFCd (Yeterian and Pandya 2010). The cingulate fasciculus (CF) connects RSP/PH to PFCd and 24ab/9 m (Mufson and Pandya 1984). The uncinate fasciculus (UF) connects TE to PFCv and also connects the rostral superior temporal gyrus to PFCvm (Petrides and Pandya 2007). Thus, white matter bundles in cortex link clustered areas that share inputs and reflect the nested organization of neocortex.

The nested model of cortical connectivity can also be seen in human and macaque imaging data (Vijayakumar et al. 2019), although it is expanded in humans. In humans, for example, the vSLF shows stronger connectivity with the ventral premotor cortex compared to monkey and it connects to more anterior Brodmann areas (BAs) 45 and 47, suggesting an evolutionary caudal–rostral extension of the vSLF that is consistent with the largest expansion of the frontal cortex in humans. Stronger prefrontal connectivity of the vSLF, along with its right lateralization, has been suggested to reflect higher cognitive functions and human motor skills such as the use of tools and social learning (Hecht et al. 2015; Thiebaut de Schotten et al. 2012). Differences also emerge in the organization of the arcuate fasciculus (AF) that follows from the expansion of the temporal cortex in humans. In monkeys, the AF connects the most caudal part of the superior temporal gyrus (area Tpt) to areas 44, 46d, 8a and the dorsal premotor cortex. In humans, the AF connects most of the temporal cortex to much of the ventral prefrontal region. Specifically, AF connects BAs 21, 22, 37, 41 and 42 of the temporal cortex, to BA 6 of the precentral gyrus and BAs 8, 9, 44 and 45 of the middle and inferior frontal gyrus (Thiebaut de Schotten et al. 2012). This reorganization of the AF has been proposed to be the anatomical foundation of language, linking posterior regions involved in the perception of words based on visual and auditory stimuli to more anterior motor areas controlling the execution of orofacial movements (Aboitiz and Garcia 2009; Thiebaut de Schotten et al. 2012).

## Shared sub-cortical projections of connected posterior-frontal areas

Besides the nested architecture, frontal cortical areas have an organized pattern of connectivity with the striatum and MD thalamus. Lateral prefrontal areas around the principal sulcus project to the dorsal striatum and the lateral MD thalamus, and ventromedial prefrontal and caudal orbital areas project to the ventral striatum and the medial MD thalamus. Areas intermediate between these locations, on coronal sections, project to intermediate locations in each structure. In addition to these two principles, it has also been shown that posterior areas that connect to the frontal areas just discussed, tend to send projections to similar striatal and thalamic regions. Therefore, within the posterior–frontal circuits, we have identified in the cluster analysis (Fig. 2), connected clusters send projections to overlapping sub-cortical areas in the striatum (Yeterian and Van Hoesen 1978) and thalamus (Selemon and Goldman-Rakic 1988). It is not the case that any two connected cortical areas have shared sub-cortical projections. However, connected posterior and frontal nodes defined by our cluster analysis have shared sub-cortical projections.

**Fig. 4:**
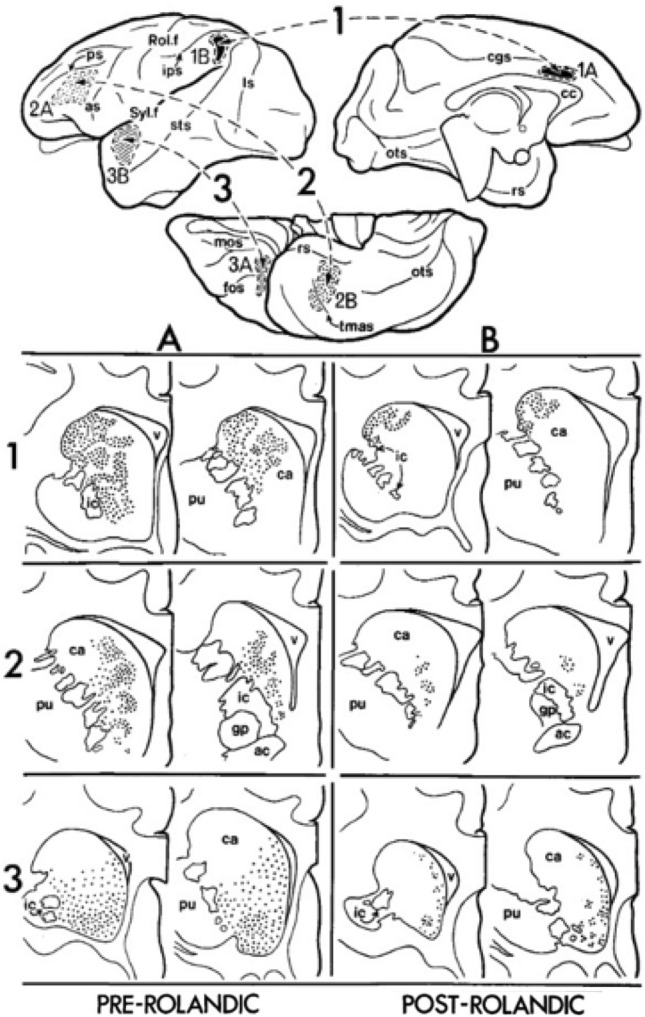
Overlapping projections of pre- and post-rolandic connected areas to the striatum. The upper figure shows the tracer injection sites into three pairs of connected pre- and post-rolandic cortical regions in a lateral, medial, and ventral view of the cortex. The bottom figure illustrates the resulting pattern of cell labeling in the striatum (shown in coronal sections). Generally, connected cortical regions project to similar regions of the striatum. Parieto-prefrontal areas (labeled 1) project to the dorsal portion of the caudate, temporal-ventral prefrontal areas (labeled 2) project to the middle/ventral caudate, and temporal- orbitofrontal areas (labeled 3) project to the ventral caudate. Figure from Yeterian and Van Hoesen (1978). Columns labeled A and B correspond to projections from frontal (A) and parietal/temporal (B) areas respectively

Most of the connected areas within the nested cortical circuits we have identified have been shown to have overlapping projections to the striatum and thalamus (e.g., Fig. 4). Entorhinal cortex, perirhinal cortex, and the amygdala project to caudal orbitofrontal and ventromedial prefrontal cortex and also to the ventral striatum and the medial portion of the MD thalamus (Friedman et al. 2002; Kondo et al. 2005; Lavenex et al. 2002; Munoz and Insausti 2005; Russchen et al. 1987; Saunders et al. 2005). Caudal orbitofrontal and ventromedial prefrontal cortex also project to the same locations in these sub-cortical areas. Similarly, area Opt/7a in parietal cortex projects to dorsolateral prefrontal areas 46 and 8 and also projects to the dorsal striatum and the lateral MD nucleus, as does area 46 (Selemon and Goldman-Rakic 1985, 1988). Area TE, which projects to PFCv, projects to a medial region of the MD that is consistent with the region to which PFCv projects (Barbas et al. 1991; Webster et al. 1993). Area TE also sends projections to the mid-striatum that overlap with the ventral prefrontal inputs (Gerbella et al. 2016; Yeterian and Van Hoesen 1978). Areas within the dorsal parietal cluster (PARd), which project to PMd, send overlapping projections into thalamic and striatal areas to which PMd projects. Specifically, area PEc, which provides visual input to PMd (Caminiti et al. 1996), sends projections to lateral MD and the central lateral (CL; which is just lateral to the anterior MD) thalamus (Yeterian and Pandya 1985), which overlap projections of the PMd (Akert and Hartmann-von Monakow 1980). PEc also sends projections to the anterior striatum (Yeterian and Pandya 1993) which may have overlap with the PMd projections (Calzavara et al. 2007), although the parietal projections may be more lateral. Parietal area Opt/7a and prefrontal areas 46 and 8 also show reciprocal connections with overlapping territories in more posterior thalamic nuclei, in particular the lateral and medial pulvinar (Asanuma et al. 1985; Barbas et al. 1991; Darian-Smith et al. 1999; Romanski et al. 1997). Similarly, parietal area PEa and the PMd show connections with similar regions in the lateral posterior nucleus (Cappe et al. 2009, 2007; Rouiller et al. 1998). Medial parietal area PGm, projects dorsally into the striatum, overlapping the PMd projections (Yeterian and Pandya 1993). Area PGm also projects lightly to MD and to CL. The ventral parietal areas (PARv) have similar overlap in CL and ventral MD with PMv areas (Akert and Hartmann-von Monakow 1980; Yeterian and Van Hoesen 1978), and PARv and PMv send projections to overlapping regions in the mid-lateral striatum (i.e., putamen (Gerbella et al. 2016)).

The somatomotor areas also have shared projections into the thalamus and striatum (Kunzle 1976, 1977), although they also have divergent projections. Both primary motor cortex (M1) and primary sensory cortex (S1) project to the ventroposterior inferior (VPI) thalamus. They may also have overlapping projections in the ventroposterior medial (VPM) thalamus. There is also an overlapping projection into the intralaminar centromedian (CM) nucleus. In addition, both areas project to overlapping regions of the putamen, in the lateral portion of the striatum (Kunzle 1977).

Thus, posterior and frontal areas that share reciprocal connectivity in the nested architecture, send projections to overlapping regions of the striatum and MD and adjacent regions of the thalamus. Although posterior and frontal areas share these sub-cortical projections, there are differences. Overlap in striatal projections tends to be more substantial than overlap in thalamic projections. To some extent, this follows from the fact that striatal projections tend to be more diffuse than thalamic projections. In addition, posterior areas tend to have smaller projections to both the anterior striatum and MD thalamus than prefrontal areas. Additionally, the MD thalamus sends reciprocal connections back to prefrontal areas, but not to the posterior areas.

## Topological organization of connections between frontal cortex and the striatum and thalamus

**Fig. 5:**
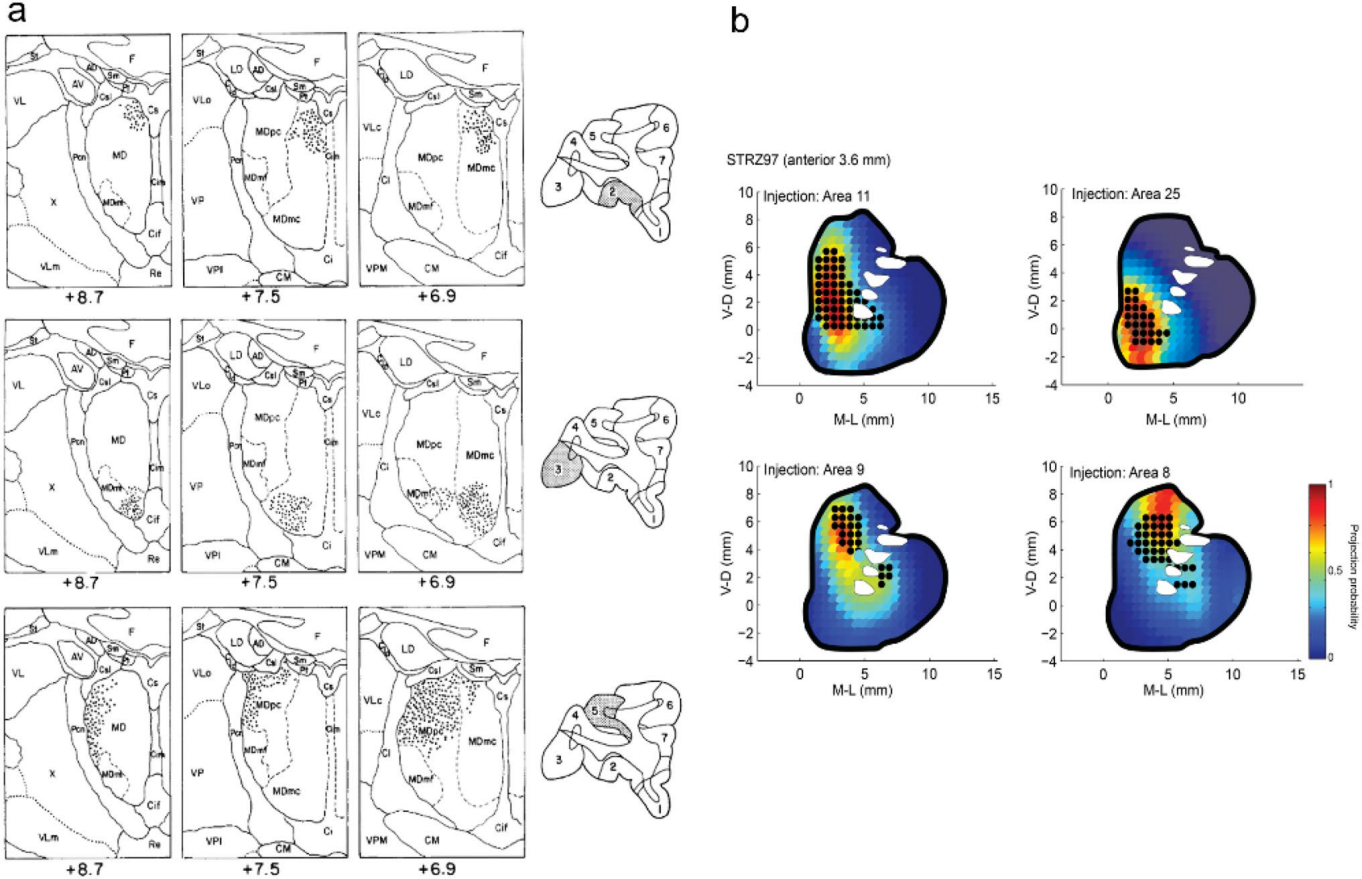
Topographical organization of connectivity between prefrontal regions and the MD thalamus and the striatum. **a** Schematic illustration of the pattern of cell labeling in the MD thalamus after tracer injections into different prefrontal regions. Caudal OFC (top) projects to the medio-dorsal MD thalamus, while PFCv (middle) and PFCd (bottom) project to the ventromedial and dorsolateral MD thalamus. **b** Predicted (heat map) and actual (black dots) projections into the striatum after tracer injection into four prefrontal regions. Anterior OFC (area 11) and PFCvm (area 25) project to the middle and ventral portion of the striatum, respectively (top panels). PFCd (areas 8 and 9) project to middle and dorsal portions of the striatum. All panels show coronal sections; a adapted with permission from Goldman-Rakic and Porrino (1985); **b** adapted from Averbeck et al. (2014)

Several groups have described a topographical organization of prefrontal projections into the striatum (Averbeck et al. 2014; Ferry et al. 2000; Haber et al. 2006) and MD thalamus (Barbas et al. 1991; Giguere and Goldman-Rakic 1988; Goldman-Rakic and Porrino 1985; Ray and Price 1993; Siwek and Pandya 1991). The primary features of this organization were apparent in early studies with less sensitive techniques, and therefore reflect the dominant connectivity of these systems (Nauta 1964). Thus, ventromedial and caudal–orbital–prefrontal cortex project to the medial magnocellular portion of the MD thalamus (Fig. 4a) and the ventral striatum (Fig. 5b—area 25). Area 46, on the other hand, and to the more lateral, parvocellular portion of the MD thalamus (Fig. 5a). Area 8, which is caudal to area 46, projects most laterally into the MD thalamus, into the multiform area, and dorsally into the striatum (Fig. 5b). Areas between these two poles, when viewed as a coronal section through prefrontal cortex, including the ventrolateral prefrontal cortex (e.g., area 12 l) or the dorsal cingulate (e.g., 9 m), project to intermediate regions of the striatum and the MD thalamus. Within the MD thalamus there is also a dorsal–ventral organization, with dorsolateral prefrontal areas projecting to the dorsal MD nucleus, and ventrolateral prefrontal areas, projecting to the ventral MD nucleus (Fig. 5a) (Barbas et al. 1991; Goldman-Rakic and Porrino 1985; Siwek and Pandya 1991). The pulvinar also shows topographical connectivity with prefrontal cortex (Romanski et al. 1997). The central and lateral medial pulvinar is reciprocally connected with lateral and orbital prefrontal areas and the medial portion of the medial pulvinar projects to medial and ventral–medial prefrontal areas.

Dorsal premotor cortex, which is just caudal and medial to dorsal prefrontal cortex, also sends projections to the MD thalamus (Akert and Hartmann-von Monakow 1980). Dorsal premotor cortex projects to lateral MD, perhaps just lateral to where area 46 projects, and also projects into the most dorsal portion of the striatum (Calzavara et al. 2007). Ventral premotor cortex also projects to the ventral portion of the lateral MD (Akert and Hartmann-von Monakow 1980; Bruni et al. 2017) and to the mid-striatum, primarily into the putamen (Gerbella et al. 2016).

The organization of prefrontal inputs to the striatum is highly regular and consistent and can be quantified with a simple linear model (Averbeck et al. 2014). This model characterized the projection zone into the striatum using the 2-D coordinate of the injection site in prefrontal cortex. Specifically, the coordinates used for prediction were the location on a coronal section, relative to the cortical crown and the anterior–posterior location. The model showed that a regular, cylindrical coordinate system exists that organizes the connectivity between prefrontal cortex and the striatum. Furthermore, nearby injection locations on the cortical surface project to nearby locations in the striatum (Fig. 5b). A similar model could be developed for projections into the thalamus, given their regular organization.

## Cortico-basal ganglia–thalamocortical loops

**Fig. 6:**
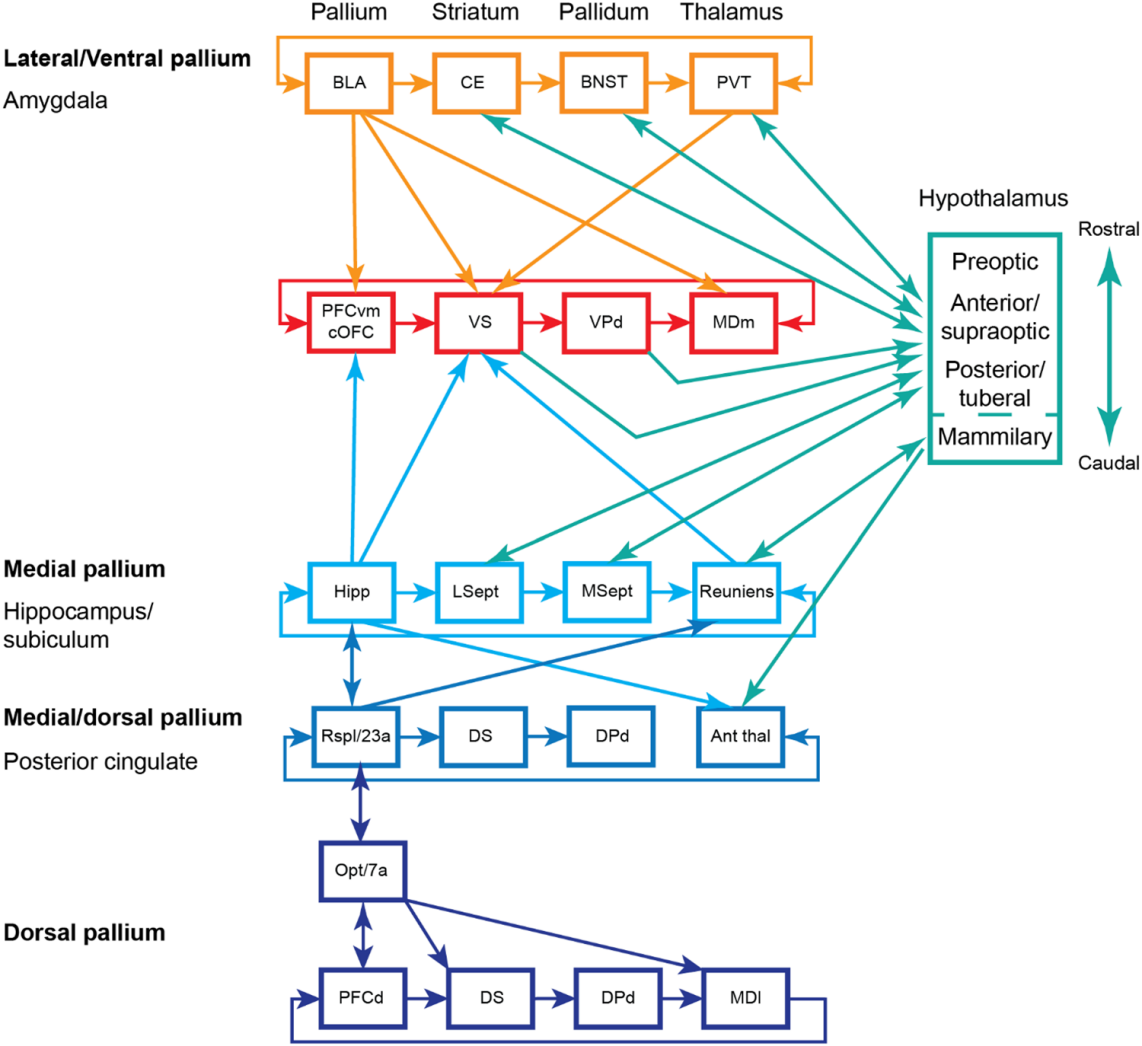
Schematic illustration of example pallial (cortical)–striatal–pallidal–thalamocortical circuits. Posterior areas, (Opt/7a, BLA) project to anterior nodes, and also to sub-pallial nodes, to which the same anterior nodes project. Note the medial and ventral–lateral pallial areas are interconnected with the hypothalamus, whereas the dorsal pallium is not. *BLA* basal-lateral amygdala, *CE* central nucleus of the amygdala, *BNST* basal nucleus of the stria terminalis, *PVT* paraventricular nucleus, *PFCvm* ventromedial prefrontal cortex, *cOFC* caudal orbital frontal cortex, *VS* ventral striatum, *VP* ventral pallidum, *MDm* medial portion of the medial dorsal thalamus, *Hipp* hippocampus, *LSept* lateral septum, *MSept* medial septum, *Rspl* retrosplenial, *DS* dorsal striatum, *DPd* dorsal pallidum, *Ant Thal* anterior thalamic nuclei, *PFCd* dorsolateral prefrontal cortex, *MDl* lateral portion of the medial dorsal thalamus.

As has been reviewed previously (Alexander et al. 1986; Haber 2016; Middleton and Strick 2000), the topographic organization of frontal connectivity in the striatum continues through the basal ganglia output nuclei, which themselves project back to thalamic nuclei that project back to the frontal areas from which they receive inputs (Fig. 6). These have been labeled the cortical–basal ganglia–thalamocortical (CBGTC) loops. As just noted, there are inputs from the posterior areas to the thalamic regions that receive inputs from the prefrontal areas to which the posterior areas project (Fig. 6). There are also reciprocal connections between thalamic and prefrontal areas. But there are not reciprocal connections between these thalamic areas and the posterior areas. For example, area Opt/7a has a projection to the lateral MD thalamus, but the lateral MD thalamus does not project back to area Opt/7a. Area 46, however, both projects to and receives reciprocal projections from the lateral MD. Similarly, the amygdala projects to the medial MD thalamus, but does not receive a reciprocal projection from the MD thalamus, whereas ventromedial prefrontal cortex (areas 14/25/32) projects to and receives reciprocal projections from the medial MD.

The CBGTC loops were originally defined for prefrontal and motor circuits through the basal ganglia. However, subsequent work expanded these loops to hippocampal and amygdala circuits (Alheid and Heimer 1988; Swanson 2000). In this framework, the central nucleus of the amygdala (CE) and the lateral septum are considered striatal nodes, and the basal nucleus of the stria terminalis (BNST) and medial septum are pallidal nodes. This work emphasized the connections of the neocortical circuits with thalamus and brainstem areas, and the amygdala and hippocampal circuits with the hypothalamus (Fig. 6). More recent developmental gene expression work suggests that the CE and BNST develop from the medial and lateral ganglionic eminence, as do the striatum and pallidum. However, the CE and BNST are composed of combinations of cells from each field, and therefore they may operate in parallel rather than in series (Bupesh et al. 2011), and their homology to the striatum and pallidum may be more complex.

## Organization of the telencephalon from developmental and comparative perspectives

**Fig. 7:**
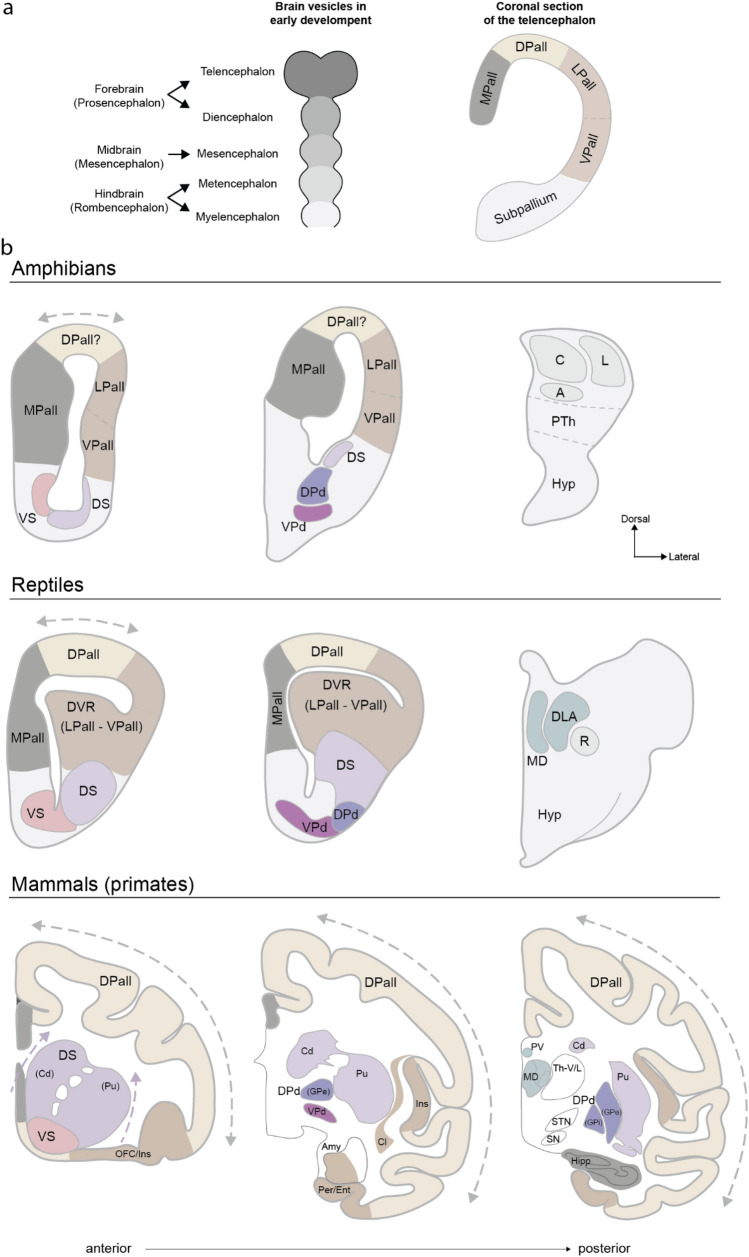
Organization of pallial and non-pallial territories in the forebrain of vertebrates. a In early development of vertebrates, the embryonic central nervous system is distinguished by the progressive elaboration of the neural tube into five vesicles (left panel). Posteriorly, the metencephalon, myelencephalon and mesencephalon, give rise to the brain stem. Anteriorly, the telencephalon and the diencephalon give rise to the forebrain. The telencephalon can be further divided into the pallium (dorsally) and sub-pallium (ventrally). Based on converging evidence from anatomical, developmental, and gene expression studies, the pallium of vertebrates, at least amniotes, can be further divided into a dorsal, medial, lateral, and ventral portion (right panel). **b** Coronal sections of vertebrate brains showing the distribution of pallial and sub-pallial sectors. In amphibians and reptiles most of the pallium is composed of the medial, lateral, and ventral pallium. The dorsal pallium is less expanded in non-mammalian vertebrates, while in mammals it represents most of the cortex (neocortex). The largest expansion of the dorsal pallium in mammals, in particular in primates, occurred in parallel with a ventro-dorsal expansion of the striatum and a re-arrangement of thalamic nuclei, which expanded medio-laterally. In mammals the lateral pallium corresponds to the claustro-insular complex, the orbitofrontal cortex, and the perirhinal/entorhinal cortex. The ventral pallium consists of the olfactory cortex and part of the amygdala, whereas the medial pallium corresponds to the hippocampal complex and part of the cingulate cortex. *MPall* medial pallium, *DPall* dorsal pallium, *LPall* lateral pallium, *VPall* ventral pallium, *VS* ventral striatum, *DS* dorsal striatum, *DPp* dorsal pallidum, *VPd* ventral pallidum, *GPe* globus pallidus pars externa, *GPi* globus pallidus pars interna, *Cd* caudate, *Pu* putamen, *DVR* dorsal ventricular ridge, *C* central thalamus, *L* lateral thalamus, *A* anterior thalamus, *PTh* prethalamus, *Hyp* hypothalamus, *MD* medio-dorsal thalamus, *DLA* dorsolateral anterior thalamus, *R* nucleus rotundus, *PV* paraventricular thalamic nucleus, *Th-V/L* ventrolateral thalamus, *STN* subthalamic nucleus, *SN* substantia nigra, *Hipp* hippocampus, *CL* claustrum, *Ins* insular cortex, *Amy* amygdala, *OFC* orbitofrontal cortex, *Per/Ent* perirhinal and entorhinal cortex

The work discussed above suggests that the general pattern of anatomical connectivity of the primate forebrain can be understood using a few principles. In the next section, we suggest that this organization reflects the evolution and developmental processes that have led and lead to the connectivity in the adult primate forebrain. Our hypothesis is that the topological organization of the forebrain outlined above reflects the evolutionary expansion of the circuits. Specifically, the cortical areas in primates that project to the ventral striatum, including the ventromedial and caudal–orbital–prefrontal cortex, hippocampus, entorhinal cortex, and amygdala, are homologous to areas that made up most of the non-mammalian pallium. Thus, the circuits through the ventral striatum may represent ancestral, conserved circuitry. The dorsal pallium and the corresponding dorsal striatum and dorsal pallidum make up a smaller proportion of the forebrain in non-mammalian vertebrates (Fig. 7a, b). The neocortex in mammals and the corresponding dorsal striatum, dorsal pallidum, and lateral portions of the MD thalamus, expanded in a topological way, such that as neocortex expanded, it projected into topologically expanding territory in the striatum and thalamus. The striatum therefore expanded across evolution from ventral to dorsal and the MD thalamus expanded from medial to lateral, as the pallium expanded from being mostly medial and ventral/lateral pallial areas to being dominated by the expanded dorsal pallial, neocortical areas (Fig. 7b). This expansion was topological because it preserved adjacency relationships, which are reflected in the connectivity gradients reviewed above. The pulvinar complex in the thalamus has also expanded in parallel with the expansion of neocortical areas and shows a topographical pattern of connectivity with cortex (Baldwin et al. 2017; Chalfin et al. 2007; Homman-Ludiye et al. 2018, 2020). Thus, the organization of the connectivity described above reflects the gradual expansion of the forebrain across the vertebrate lineage leading to large-brained primates. This hypothesis does not account for the nested architecture or the matched sub-cortical projection targets of connected posterior and frontal cortical areas. To account for these one has to assume additionally that the neocortex, excluding A1 and V1, expanded from the inside out, starting with S1. As areas were added posterior and anterior to S1, they maintained connectivity, reflected in the major white matter bundles, and also maintained shared sub-cortical connectivity. The evidence for this second assumption is currently less clear, but we consider it below.

## Tripartite organization of the pallium

**Fig. 8:**
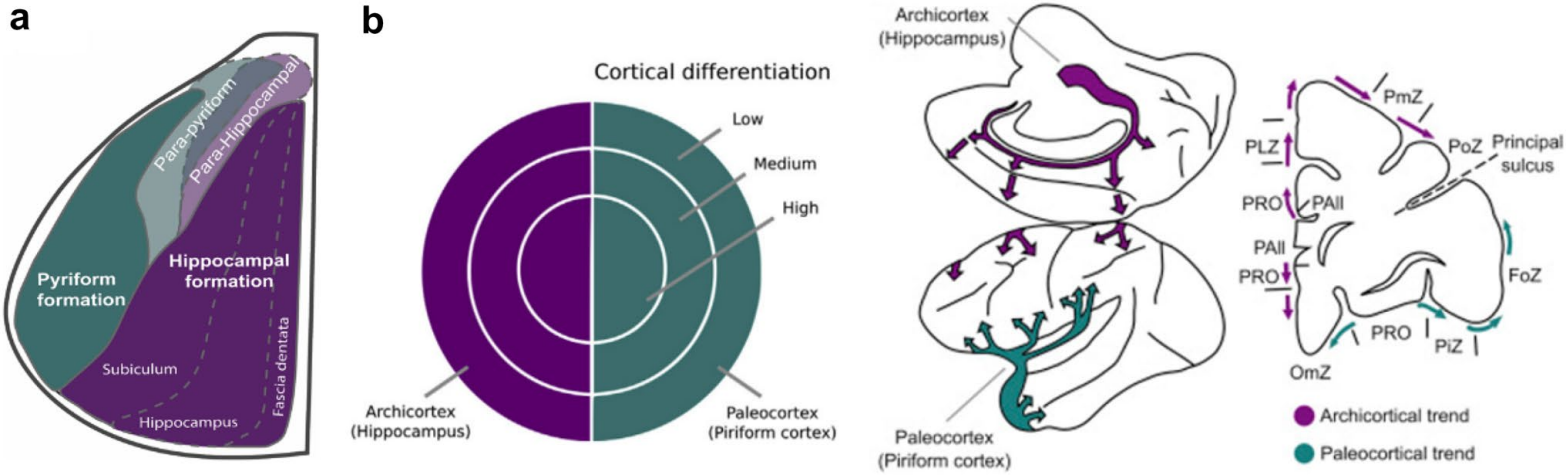
Tripartite model of pallial organization and the dual-origin hypothesis. **a** Dorsal projection of the reptilian pallium showing three distinct neural territories, where the para-pyriform and para-hippocampal are lumped as dorsal pallial. Medially, the hippocampal formation (or archicortex) processes spatial information, and laterally the pyriform formation (or paleocortex) processes olfactory information. **b** The dual-origin hypothesis proposes that mammalian cortex can be traced back to the archicortex and the paleocortex as a result of their progressive differentiation in terms of cytoarchitecture and laminar elaboration. Green and purple arrows illustrate the paleo-cortical and the archicortical trends in the macaque cortex. a redrawn from Dart (1934); **b** with permission from Goulas et al. (2019)

The vertebrate pallium is a term that refers to the mammalian cortex, as well as the homologous structures in non-mammalian vertebrates. The pallium and sub-pallium, which correspond to the cortex and basal ganglia, comprise the telencephalon (Fig. 7a). It has long been suggested, originally in comparative architectonic studies, that the pallium can be divided into regions in vertebrates (Abbie 1940, 1942; Dart 1934; Pandya and Yeterian 1985; Sanides 1970). Early work in reptiles (Dart 1934) and mammals (Abbie 1940, 1942) suggested that the pallium could be divided into a medial, hippocampal component and a lateral, pyriform component. Between these were transition areas which were neo-pallial (neocortical in mammals) (Fig. 7b, Fig. 8a). The neo-pallial areas derived from either the medial or lateral pallial areas, to which they were adjacent. These early studies, therefore, divided the pallium into three areas. Medial pallial areas corresponding to the hippocampus are known as archipallial. Dorsal pallial areas corresponding to the neocortex are known as neo-pallial. And lateral pallial areas corresponding to pyriform cortex are known as paleo-pallial. This is referred to as the tripartite model of pallial organization (Puelles et al. 2000, 2017).

A line of research known as dual origin theory has continued to develop this model (Pandya et al. 2015; Pandya and Yeterian 1985; Sanides 1970). It was developed on the basis of cytoarchitectonic data in humans and macaques (Sanides 1964). The hippocampal component in the anterior cingulate, just dorsal to the corpus callosum is agranular, three-layer cortex, as is olfactory/pyriform cortex. As one moves dorsal from the anterior cingulate, or anterior and lateral from olfactory cortex, the cortex becomes increasingly laminar or dysgranular. On the lateral surface the cortex continues to become more laminar, until the two trends meet on the lateral surface of prefrontal cortex. In the macaque, this happens at the bottom of the principal sulcus, and in humans at the bottom of the inferior frontal sulcus. Area 8 in dorsolateral prefrontal cortex is the most granular. Note that this model was developed in prefrontal cortex, and therefore it focuses on expansion from medial and ventrolateral pallial areas, ignoring posterior, primary sensory, dorsal pallial areas, including primary auditory, visual and somatosensory regions (Halley and Krubitzer 2019).

This organization is reflected in the cluster analysis of frontal areas (Fig. 2a). The clustering algorithm was originally developed for phylogenetics. The Ia/13 cluster adjoins olfactory cortex, and therefore may reflect the most primitive lateral pallial area included in our analysis. The PFCvm cluster, followed by the aOFC cluster, may have originated, in sequence, as cortex expanded. The dorsolateral areas, PFCv and PFCd, appeared last. The cingulate clusters (CMA and 24ab/9 m) represent the medial trend. The motor areas developed anteriorly, starting from M1, expanding into SMA and PMv areas, followed by PMd.

The dual origin model has been further developed and used to characterize a hierarchical connectional architecture in prefrontal cortex (Barbas and Pandya 1989; Barbas and Rempel-Clower 1997). It has been shown, for example, that connectivity between areas is strongest when they have the same level of architectonic differentiation (Barbas and Pandya 1989). Furthermore, connections in sensory cortex are often defined as feed-forward, originating in layer III and terminating in layer IV, or feedback, originating in layers V/VI and terminating in layer I/VI. A similar arrangement is seen in frontal areas. Projections from agranular (limbic) areas to increasingly granular (eulaminate) areas are feedback, and connections from eulaminate areas are feed-forward. Other authors have shown that the dual origin theory predicts large-scale patterns of connectivity between sensory areas and frontal cortex (Goulas et al. 2019). These patterns are largely consistent with the cortical organization we have set forth.

The cytoarchitectonic gradients in prefrontal cortex also parallel gene expression profiles (Burt et al. 2018). It has been shown, using structural magnetic resonance imaging that the agranular areas, which are low in myelin, have unique patterns of gene expression relative to the granular areas (Burt et al. 2018). Related work has shown that human primary and higher-order visual areas also show gene expression gradients related to organization across or within areas, and these genetic gradients parallel cortical thickness and myelination (Gomez et al. 2019, 2021). Thus, modern imaging and genetic techniques are revealing organizational patterns similar to those identified earlier with architectonic analysis.

The tripartite model of pallial organization remained dominant for most of the 20th century. The exact boundaries between areas, however, are often difficult to identify with cytoarchitectonics and exact homologies across species are contentious (Striedter and Northcutt 2020). The recent advent of developmental gene expression studies, that use the expression of transcription factors to identify boundaries between regions (O’Leary et al. 2007; Tosches 2017; Tosches et al. 2018), has improved the identification of field homologous areas, which are areas that have a shared developmental origin, across species. These studies have supported the division of the pallium into medial, dorsal, and lateral regions. However, some authors have further suggested that the lateral region should be subdivided into lateral and ventral regions, with the lateral region corresponding to the claustrum and insula, and the ventral region corresponding to the pyriform region and the pallial amygdala (Puelles et al. 2019). Other authors have gone beyond this and suggested that the pallium can be divided into 6 regions, based on developmental gene expression data (Desfilis et al. 2018). The additional areas, however, are subdivisions of the original three areas.

The dorsal pallium is the dominant cortical structure in mammals, including an additional expansion in primates (Chaplin et al. 2017; Finlay 2009). Some authors have suggested that all vertebrates have a dorsal pallial region (Suryanarayana et al. 2017, 2021). However, there is disagreement on this point. Homologues of the dorsal pallium are often defined on the basis of several criteria. Specifically, dorsal pallial homologues should receive unimodal sensory projections from the dorsal thalamus, they should be positioned between the medial and the ventral–lateral pallium, they should not receive direct olfactory projections, and they may send projections to sub-cortical structures like the tectum. Birds have a region homologous to the dorsal pallium known as the wulst, which receives inputs from the dorsal lateral geniculate nucleus. The wulst may also have columnar organization, which is a defining feature of neocortex (Stacho et al. 2020). Reptiles also appear to have a small dorsal pallial region that receives unimodal visual and somatosensory input from the dorsal thalamus (Medina 2007), and a possible dorsal pallial homologue has also been identified in lamprey (Suryanarayana et al. 2017, 2021). The dorsal pallium has not been consistently identified in fish and amphibians, however (Striedter and Northcutt 2020). These authors have argued that the dorsal pallium arose with amniotes, which includes birds, reptiles, and mammals. The pallial region that has expanded massively to become the neocortex in mammals, therefore, was at most small and possibly absent prior to the advent of amniotes. The medial and lateral pallium, however, have been consistently identified across vertebrates. The primitive vertebrate pallium, therefore, may have been composed of a medial, hippocampal pallium and a ventral–lateral, pyriform pallium, and perhaps a small dorsal pallium.

## Organization of sub-pallial circuits

The two dominant structures of the vertebrate sub-pallium are the striatum and the pallidum. Both structures appear to be well-preserved across vertebrates (Reiner et al. 1998). They have been defined in the lamprey, which along with hagfish define the basal vertebrate clade of jawless fish (Stephenson-Jones et al. 2011). The striatum develops from the lateral ganglionic eminence and the pallidum develops from the medial ganglionic eminence (Moreno et al. 2009). The projection neurons in both the striatum and the pallidum are inhibitory, and the medial and lateral ganglionic eminence fields produce most of the inhibitory neurons in the telencephalon, although human cortical progenitor cells also give rise to inhibitory interneurons (Delgado et al. 2022). The striatum is composed of two groups of GABAergic projection neurons referred to by various names including medium spiny neurons (MSNs) and phasically active neurons. One group, the direct pathway MSNs, expresses primarily D1 dopamine receptors and corelease substance P. The second group, the indirect pathway MSNs, expresses primarily D2 dopamine receptors and corelease enkephalin. Both groups of neurons have been identified in lampreys (Stephenson-Jones et al. 2011), and many other groups.

In mammals, there is a dorsal and ventral striatum, and corresponding well-defined dorsal and ventral pallidum. The dorsal striatum projects to the dorsal pallidum, and the ventral striatum projects to the ventral pallidum. Organization along the dorsal–ventral axis in the striatum is better thought of as a continuum (Voorn et al. 2004), but the dorsal-most part of the striatum and the ventral-most part have different anatomical connectivity (Haber 2016). Between the dorsal and ventral striatum, however, there is a gradient of connectivity (Averbeck et al. 2014; Haber et al. 2006), as discussed above. The dorsal and ventral pallidum are more distinct (de Olmos and Heimer 1999; Heimer 1975; Zahm et al. 1987). In primates, the dorsal pallidum is divided into an internal and external component, and each component receives inputs from a specific class of MSNs in the dorsal striatum. The direct pathway MSNs project to the internal segment of the dorsal pallidum and the indirect pathway MSNs project to the external segment of the dorsal pallidum. This distinction is not present for the ventral striatal projections to the ventral pallidum (Kupchik et al. 2015). Both direct and indirect pathway MSNs in the ventral striatum project to the ventral pallidum. This mixing of the direct and indirect pathway neurons within a single pallidal nucleus may be the case for all striatal outputs in non-mammalian vertebrates (Reiner et al. 1998). Thus, the ventral striatum to ventral pallidum pathway may reflect the ancestral condition, present in non-mammalian vertebrates, in which the dorsal or ventral pallidum is a single structure that receives inputs from both the direct and indirect pathway MSNs. The dorsal striatum, dorsal pallidum pathway, in which direct and indirect pathway MSNs project to segregated nuclei, is most clearly developed in primates.

In mammals, the dorsal striatum receives inputs from the dorsal pallium (neocortex), and the ventral striatum receives inputs from the medial and ventral–lateral pallial areas (archi and paleo-cortical areas). Thus, the expanded mammalian dorsal pallium projects to the dorsal striatum, which then projects to the dorsal pallidum, which is divided into internal and external components. The more conserved medial and ventral–lateral pallial structures, including the hippocampus and associated areas of the sub-callosal cingulate, caudal orbitofrontal cortex, olfactory cortex, and the amygdala, project to the ventral striatum, which projects to the ventral pallidum (Haber et al. 1985, 1993). This circuitry is also strongly connected to the hypothalamus (Fig. 6) and is generally consistent with the classically defined limbic system (MacLean 1952; Nauta 1958; Papez 1937). The ventral system in mammals, therefore, may be homologous to the ancestral system, preserved across the vertebrate lineage. The dorsal system, on the other hand, is at most a small component of the telencephalon in non-mammalian vertebrates. It has expanded massively, however, in mammals, reaching its largest extent in primates.

Non-mammalian tetrapods (amphibians and sauropsids) have both a dorsal and a ventral striatum, defined by downstream projections (Marin et al. 1997, 1998). The dorsal striatum projects to the pallidum and substantia nigra pars reticulata, through circuits that ultimately project to the tectum. The ventral striatum projects to the pallidum and to the hypothalamus. This dorsal–ventral difference in downstream basal ganglia projections is also seen in mammals (Averbeck and Murray 2020; Swanson 2000). However, both the dorsal and ventral striatum in amphibians receive inputs from the medial and ventral–lateral pallium, as amphibians have at most a limited dorsal pallium (Marin et al. 1998). Dorsal thalamic nuclei in amphibians also project strongly to the striatum, and less to the pallium, which differs from amniotes (Pessoa et al. 2019; Reiner et al. 1998). Dorsal and ventral striatal regions in amphibians, therefore, may differ more in their thalamic inputs than their pallial inputs. Therefore, the definition of a dorsal striatum in amphibians rests on the downstream projections and thalamic inputs, not pallial inputs.

Homologous thalamic structures are less well-known across vertebrates (Butler 1994). Based on chemoarchitectonic, topographic, and connectivity properties, the MD and paraventricular thalamic nuclei in mammals have been proposed to be the homologues of the dorsomedial and dorsolateral anterior thalamic nuclei in reptiles (Butler 1994; Davila et al. 2000; Heredia et al. 2002) (See also Fig. 7).

## Comparative evidence for the nested neocortical architecture

Less is known about comparative aspects of connectivity across the pallium, and whether the nested architecture we have defined in primates exists, in a less expanded form, in vertebrates with smaller brains. Comparative studies suggest that the small dorsal pallium present in reptiles may be composed only of primary visual and somatosensory areas (Medina 2007). Thalamic auditory areas project directly to the striatum, bypassing additional processing in the pallium. Reptiles do not appear to have a primary motor area separate from S1. Similarly, monotremes and marsupials, which are basal to placental mammals, also do not have consistently defined separate somatosensory and motor areas (Karlen and Krubitzer 2007; Lende 1963). Therefore, the M1/S1 areas, which are the core of our nested architecture, only separated into distinct structures with the advent of placental mammals. Comparative work in mammals also shows the consistent presence of A1, S1, and V1, but there is less information about the organization and connectivity of prefrontal areas, beyond the anterior cingulate (Halley and Krubitzer 2019). Therefore, the core of the nested structure in primates, composed of separate M1 and S1 areas, likely expanded from an integrated S1/M1 that had both sensory and motor properties.

With respect to parietal–frontal areas outside of the M1/S1 core, opposum’s have an area anterior to S1 that projects to the tectum and may receive visual input from primary visual cortex (Martinich et al. 2000). This circuit may relate to the parietal–frontal connections identified in the nested model. There are also direct inputs from primary visual cortex to other frontal areas (Martinich et al. 2000). Although there are rostral cingulate areas that project to the colliculus in squirrels (Baldwin et al. 2019) they do not appear to receive input from either primary (area 17) or secondary (area 18) visual cortex (Kaas et al. 1989). It is possible that higher-order areas that receive input from early visual areas, similar to dorsal parietal or temporal areas in primates, may project to frontal areas. Tree shrews, which are phylogenetically closer to primates than rodents are to primates, have parietal–frontal circuits (Remple et al. 2007). However, whether they have an incipient nested architecture is unclear. Thus, circuits connecting visual cortex to frontal motor areas have been seen in other mammals and therefore there is suggestive but limited evidence for simpler nested architectures in animals with less expanded neocortical areas.

## Conclusion

In summary, we propose that the organization of the dominant patterns of connectivity in the primate forebrain can be understood using a few principles, and these principles are the result of the evolutionary history that gave rise to the primate forebrain. The primate neocortex, outside of A1 and V1, has a nested architecture, with M1/S1 at its core. Around this are dorsal and ventral parietal-frontal and temporal-frontal circuits. Connected posterior and anterior cortical areas have shared sub-cortical targets in the striatum and the thalamus, and the striatal–pallidal and pallidal–thalamic connections maintain this organization in topological closed loops. Furthermore, there are connectivity gradients in both the striatum and thalamus, such that ventromedial and caudal–orbital prefrontal areas, and their connected temporal lobe areas, project to the ventral striatum and the medial portion of the MD thalamus. Dorsolateral prefrontal areas, and their connected dorsal parietal lobe areas, project to the dorsal striatum and the lateral portion of the MD and the laterally adjacent CL thalamus. Areas between these cortical poles have intermediate projections into both the striatum and the thalamus, in an organized and topological way.

The ventromedial and caudal orbital prefrontal areas, as well as the hippocampus and amygdala, that project to the ventral striatum, are homologous to the medial and ventral–lateral pallial areas that make up most of the non-mammalian pallium. The dorsal neocortical areas that project to the dorsal striatum are massively expanded in primates, and they are small in the non-mammalian pallium. Therefore, as the dorsal cortex expanded across the vertebrate lineage leading to primates, the dorsal striatum and the lateral MD thalamus also expanded, topologically, such that adjacency relationships were maintained (Fig. 7). This organization is also reflected in the organization of direct and indirect pathway outputs from the striatum, which target segregated dorsal pallidal but non-disaggregated ventral pallidal areas in the primate, also topologically. In the non-mammalian vertebrates, however, the non-disaggregated organization, characteristic of the ventral striatal circuitry, predominates.

The organization of circuitry in non-primates that may provide insight into the origins of the nested architecture is less clear. However, M1 and S1 do not separate into differentiable areas consistently until placental mammals. There are also circuits that link early visual areas to frontal areas in opossums, and there are anterior cingulate areas in squirrels that project to the tectum, but visual inputs to these areas have not been reported.

While the primate forebrain is a complex structure, we suggest that it has a relatively simple organization. The organization we suggest eliminates many of the details, particularly about connectivity at the cortical level. However, this organization may capture the large-scale pattern of connectivity. Furthermore, we suggest that this organization reflects the ancestral antecedents that have evolved into the current primate forebrain.

## Data Availability

Data sharing not applicable to this article as no datasets were generated or analyzed during the current study.
